# 2-(3, 4-dihydroxybenzylidene)malononitrile as a novel anti-melanogenic compound

**DOI:** 10.18632/oncotarget.20690

**Published:** 2017-09-06

**Authors:** Bonggi Lee, Kyoung Mi Moon, Jong Seung Lim, Yeojin Park, Do Hyun Kim, Sujin Son, Hyoung Oh Jeong, Dae Hyun Kim, Eun Kyeong Lee, Ki Wung Chung, Hye Jin An, Pusoon Chun, Arnold Y. Seo, Ju-Hye Yang, Bong-Seon Lee, Jin Yeul Ma, Won-Kyung Cho, Hyung Ryong Moon, Hae Young Chung

**Affiliations:** ^1^ Molecular Inflammation Research Center for Aging Intervention (MRCA), Pusan National University, Busan, Republic of Korea; ^2^ College of Pharmacy, Pusan National University, Busan, Republic of Korea; ^3^ College of Pharmacy, Inje University, Gimhae, Gyeongnam, Republic of Korea; ^4^ Janelia Research Campus, Howard Huge Medical Institute, Ashburn, VA, USA; ^5^ Korean Medicine (KM)-Application Center, Korea Institute of Oriental Medicine (KIOM), Dong-gu, Daegu, Korea

**Keywords:** skin, melanogenesis, tyrosinase inhibitor, pigmentation, photoaging

## Abstract

Tyrosinase is a key player in ultraviolet-induced melanogenesis. Because excessive melanin accumulation in the skin can induce hyperpigmentation, the development of tyrosinase inhibitors has attracted attention in cosmetic-related fields. However, side effects including toxicity and low selectivity have limited the use of many tyrosinase inhibitors in cosmetics. We synthesized 12 novel 2-(substituted benzylidene)malononitrile derivatives and investigated their anti-melanogenic activities. Of these 12 compounds, 2-(3, 4-dihydroxy benzylidene)malononitrile (BMN11) exhibited the strongest inhibitory activity against tyrosinase (IC_50_ = 17.05 μM). In parallel with this, BMN11 treatment notably decreased alpha-melanocyte-stimulating hormone-induced melanin accumulation in B16F10, cells without toxicity and also decreased melanin accumulation in a human skin model. As a mechanism underlying the BMN11-mediated anti-melanogenic effect, docking simulation showed that BMN11 can directly bind to tyrosinase by forming two hydrogen bonds with GLY281 and ASN260 residues, and via three hydrophobic interactions with VAL283, PHE264, and ALA286 residues in the tyrosinase binding pocket, and this likely contributes to its inhibitory effect on tyrosinase. Consistently, Lineweaver-Burk and Cornish-Bowden plots showed that BMN11 is a competitive inhibitor of tyrosinase. We concluded that BMN11 may be a novel tyrosinase inhibitor that could be used in cosmetics.

## INTRODUCTION

Melanin is produced in melanocytes located in the basal layer of the epidermis of skin, and is a major determinant of skin color. Ultraviolet (UV) radiation is a well-known stimulator of melanin synthesis in epidermal melanocytes. The synthesized melanin is transferred to keratinocytes by melanocyte dendrites. Melanin accumulation in keratinocytes can make skin darker [1]. Although melanin pigmentation in the skin is a protective mechanism against UV radiation, excessive accumulation of melanin induces hyperpigmentation and even pigmentation disorders such as melasma, freckles, and senile lentigines [2]. Therefore, numerous efforts have been made to develop whitening compounds to reduce melanogenesis [3–6].

Tyrosinase is a multifunctional type-3 copper-containing enzyme. The copper-containing sites are important for tyrosinase activity [7]. This enzyme mainly uses L-tyrosine as a substrate and catalyzes two rate-limiting steps in melanogenesis: the monophenolase hydroxylation of L-tyrosine to 3, 4-dihydroxy-L-phenylalanine (*L-DOPA*), and the oxidation of *L-DOPA* to DOPA quinine [7, 8]. Thus, inhibiting tyrosinase can be an efficient strategy to reduce melanogenesis, thereby inhibiting hyperpigmentation. However, not many tyrosinase inhibitors are currently available in the field of cosmetics and medical products because of their cytotoxicity and lack of selectivity and stability [5, 9, 10]. For example, kojic acid was developed as a strong tyrosinase inhibitor and used as an anti-melanogenic compound in cosmetics, but its use was prohibited because of cytotoxicity. In addition, certain benzaldehyde and benzoate derivatives isolated from plants were identified as tyrosinase inhibitors, including anisaldehyde, benzoic acid, cinnamic acid, benzaldehyde, anisic acid, and methoxycinnamic acid isolated from the roots of *Pulsatilla cernua* [11], 2-hydroxy-4-methoxybenzaldehyde from the roots of *Mondia whitei* [12], vanillic acid and its derivatives from black rice bran [13], and *p*-coumaric acid from the leaves of *Panax ginseng* [14]. However, advanced data are lacking for their applications as anti-melanogenic agents. Thus, additional studies are necessary to find more efficient tyrosinase inhibitors with no cytotoxicity and improved selectivity and stability. In an attempt to find a novel tyrosinase inhibitor, we synthesized 12 2-(substituted benzylidene)malononitrile derivatives. Previous studies revealed that 2-(substituted benzylidene)malononitrile analogs exhibited pharmacological activities such as antimicrobial [15], anti-proliferative [16], and β–cell protective effects [17]. In this study, we examined their tyrosinase inhibitory activity using docking simulation, and *in vitro* assays using B16F10 cells and a human skin model.

## RESULTS

**Figure 1:**
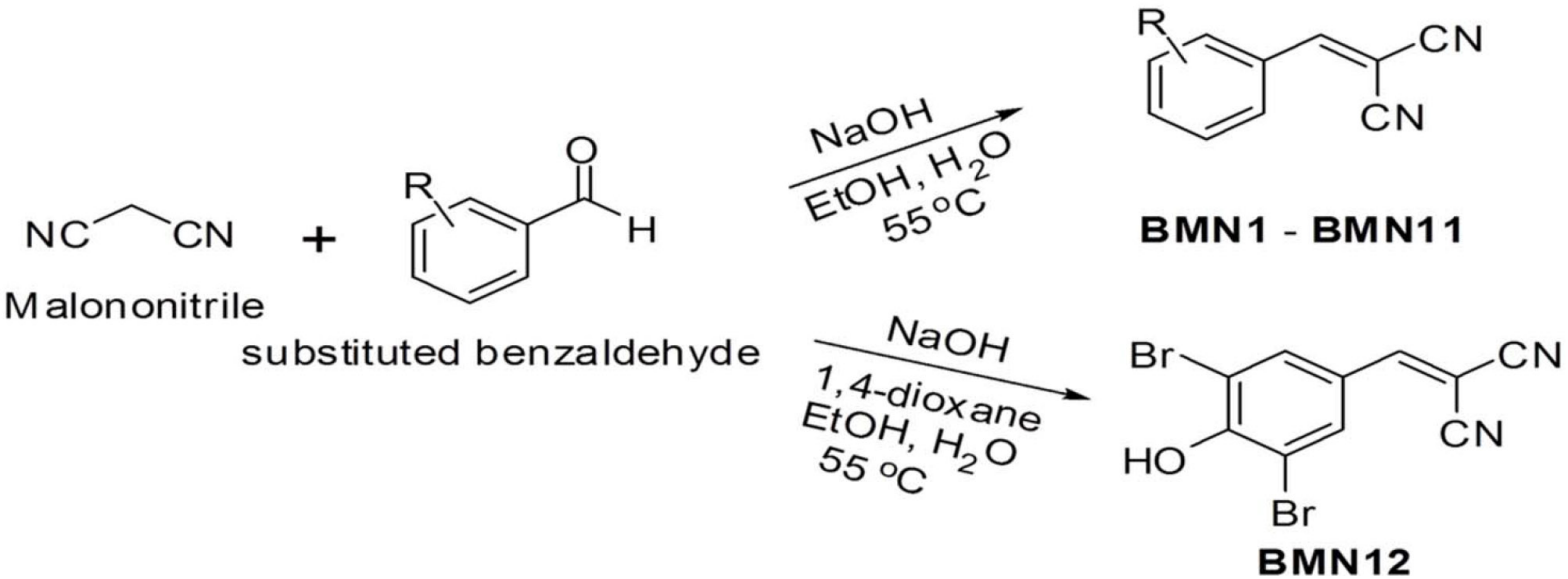
Rationale for the design of 2-(substituted benzylidene)malononitrile analogs R represents a hydroxyl group, a methoxy group, an ethoxy group or a bromo group, and may be substituted with 1 to 3 substituents. In the synthesis of BMN12, 1, 4-dioxane was added to improve the solubility of 3, 5-dibromo-4-hydroxybenzaldehyde, the starting material.

**Figure 2:**
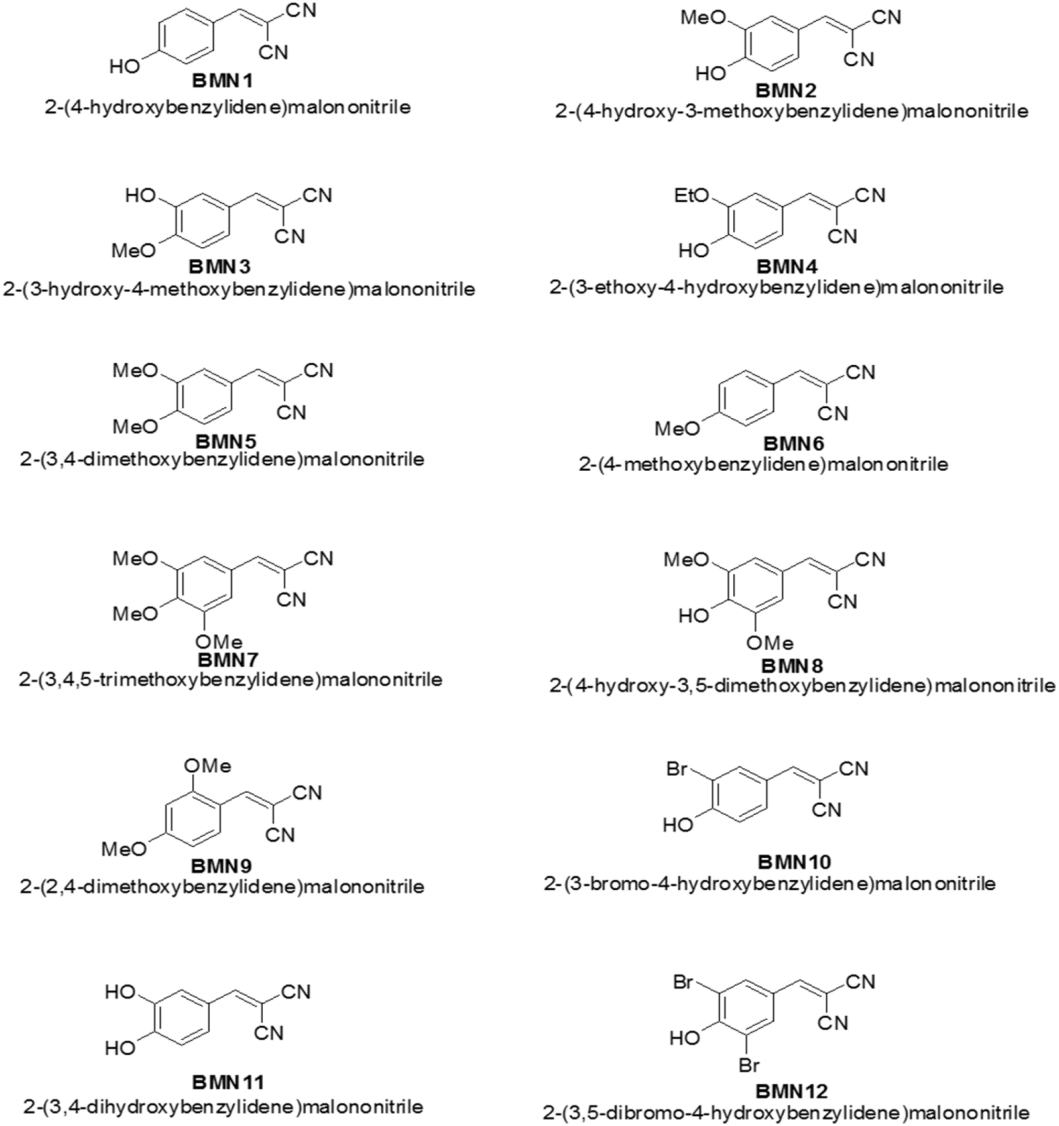
Substitution pattern of the 2-(substituted benzylidene)malononitrile derivatives Twelve 2-(substituted benzylidene)malononitrile derivatives (BMN1-BMN12) were synthesized. All the substituents of hydroxyl, methoxy, ethoxy and bromo are substituted at position 2, 3, 4 or 5 and substituted by 1, 2, or 3 substituents.

**Figure 3:**
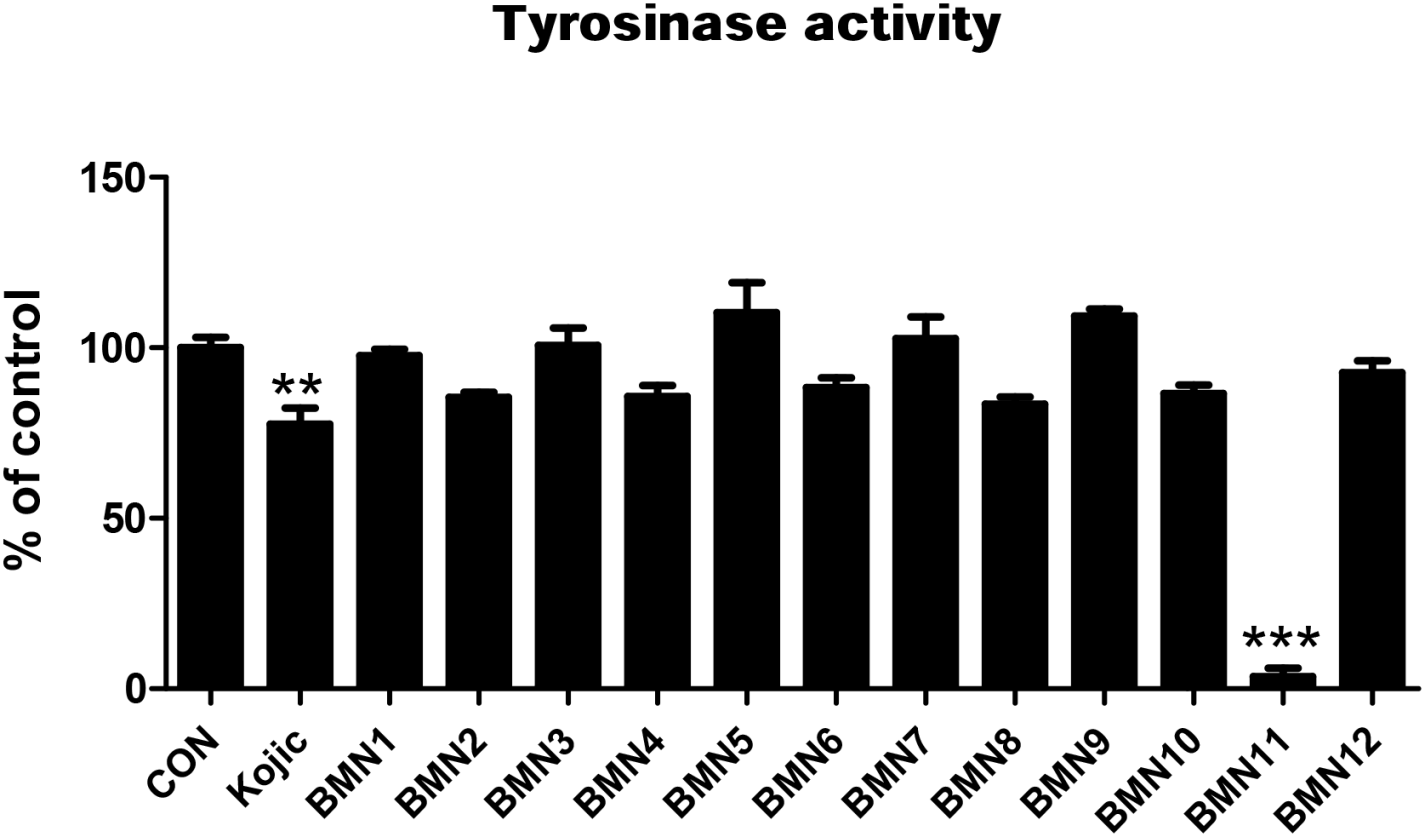
Tyrosinase inhibitory activity of BMNs The tyrosinase inhibitory activity of BMN1-BMN12 was measured using mushroom tyrosinase. BMN 1∼12 (50 μM) and kojic acid (50 μM) were loaded onto a 96-well microplate. After incubation with mushroom tyrosinase at 37°C for 15 min, dopaquinone levels were measured by spectrophotometry at 450 nm. **P < 0.01 and ***P < 0.001 compared to the control group.

Because tyrosinase regulates the rate-limiting steps of melanogenesis, suppressing this enzyme has been shown to inhibit skin pigmentation [18]. In an attempt to find effective tyrosinase inhibitors, we synthesized 2-(substituted benzylidene)malononitrile derivatives (Figure 1 and Figure 2) and investigated their anti-melanogenic activity. We used kojic acid as a positive control. Kojic acid has been shown to chelate copper at the active site of tyrosinase and suppress its activity [18]. To compare the direct tyrosinase inhibitory activity of BMNs with that of kojic acid, we performed a mushroom tyrosinase activity assay in test tubes. The data showed that of the 12 compounds tested, only 2-(3, 4-dihydroxybenzylidene)malononitrile (BMN11) exhibited tyrosinase inhibitory activity (Figure 3). We further examined the concentration-dependent inhibitory effect of BMN11 on tyrosinase, and calculated its IC_50_ values (Table 1). Data showed that the IC_50_ value for kojic acid was 36.68 μM, whereas that of BMN11 was 17.05 μM (Table 1), indicating that BMN11 is a strong tyrosinase inhibitor.

**Table 1:** IC_50_(μM) values for BMNs

Compounds	Concentration(μM)	Tyrosinase Inhibition(%)	IC_50_(μM)
BMN1	50	18.00	Not determined
	100	34.07	
	200	37.18	
BMN2	50	21.10	145.7026
	100	37.16	
	200	65.89	
BMN3	50	6.04	Not determined
	200	11.83	
	500	14.25	
BMN4	50	19.023	186.1824
	100	29.428	
	200	53.421	
BMN5	50	3.068	Not determined
	200	3.626	
	500	5.718	
BMN6	50	33.90	186.3461
	100	44.28	
	200	50.11	
BMN7	50	3.18	Not determined
	200	13.56	
	200	14.92	
BMN8	50	5.08	Not determined
	200	8.99	
	500	9.78	
BMN9	50	6.656	Not determined
	100	6.808	
	200	7.248	
BMN10	50	11.50	Not determined
	100	23.78	
	200	41.94	
BMN11	2	14.73	17.05211
	5	17.47	
	10	27.05	
	20	59.24	
BMN12	100	8	Not determined
	200	12.55	
Kojic Acid	10	9.32	36.6835
	20	28.14	
	30	40.60	
	40	54.66	

**Figure 4:**
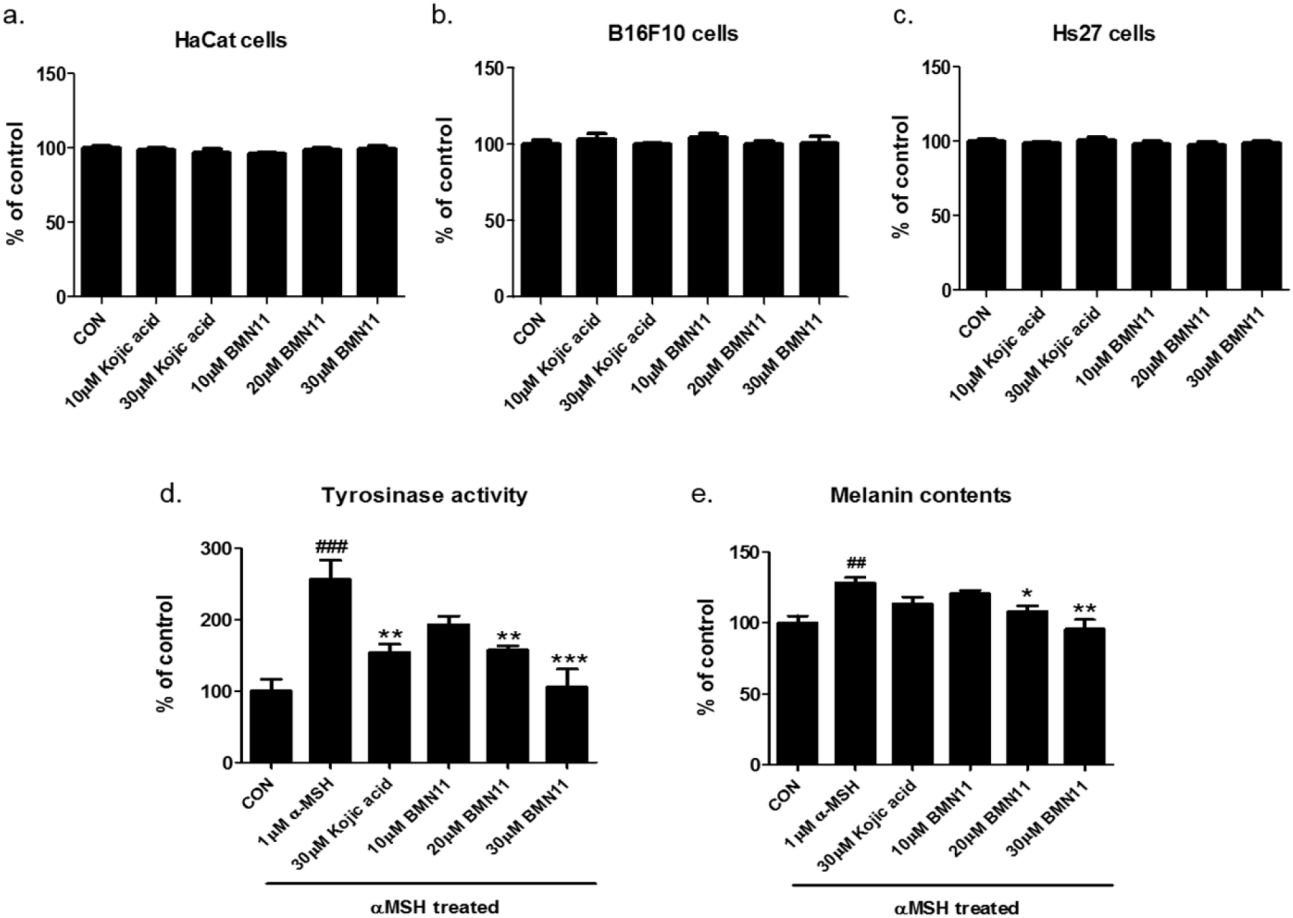
Inhibition of melanin accumulation by BMN11 in B16F10 cells **(a-c)** Cell viability post BMN11 treatment. (a) HaCat, (b) B16F10, or (c) Hs27 cells were exposed to BMN11 at various concentrations for 48 h, as shown in this figure. The MTT analysis was then performed. The results are shown as the percentages relative to the non-treated group (n = 5/group). **(d-e)** αMSH–induced tyrosinase activity and melanin accumulation in B16F10 cells post BMN11 treatments. B16F10 cells were pre-treated with BMN11 or kojic acid for 2 h. The cells were exposed to αMSH (1 μM) for 48 h to induce melanogenesis (n = 4/group). (d) Tyrosinase activity and (e) melanin content were measured using a microplate reader (n = 4/each group). The data are expressed as means ± SEM. ^##^*P* < 0.01 and ^###^*P* < 0.001 compared with the non-treated control group. *P < 0.05, **P < 0.01, and ***P < 0.001 compared to the αMSH-exposed group.

We investigated whether BMN11 inhibits melanogenesis in B16F10 cells by measuring tyrosinase activity and melanin content post treatment with alpha-melanocyte-stimulating hormone (αMSH), which is a well-known melanogenesis inducer. We first tested whether BMN11 has cytotoxicity *in vitro*. While BMN11 showed no cytotoxicity in cell lines including HaCat (human keratinocyte), B16F10 (mouse melanoma), and Hs27 (human fibroblast) up to 30 μM (Figure 4a), αMSH-induced tyrosinase activity (Figure 4b) and melanogenesis (Figure 4c) were notably decreased by BMN11 treatment in a concentration-dependent manner, suggesting that BMN11 efficiently inhibits αMSH-mediated melanogenesis.

**Figure 5:**
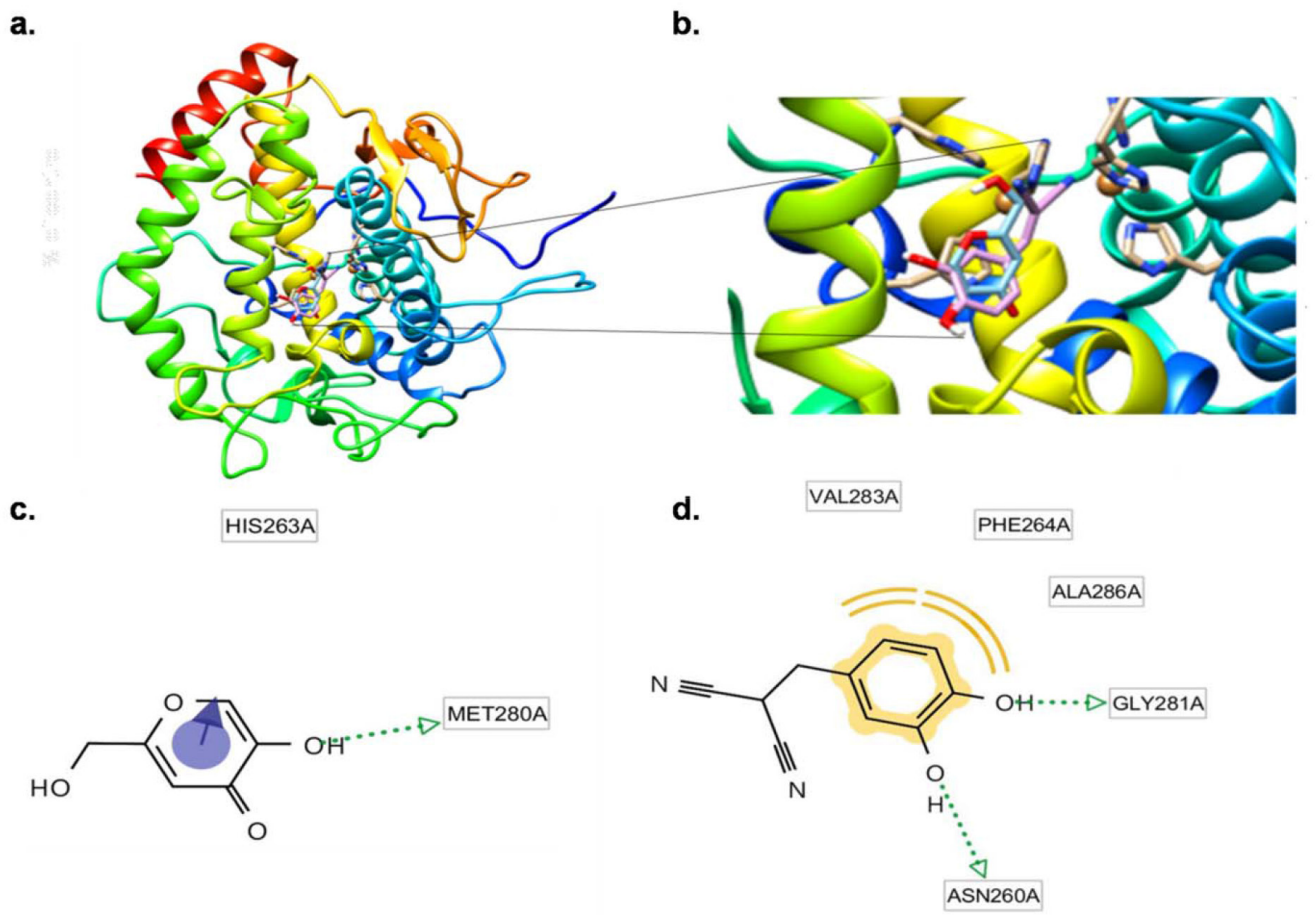
Direct binding of BMN11 to tyrosinase **(a-d)** Protein docking simulation between mushroom tyrosinase and BMN11. (a) Predicted 3D structure of mushroom tyrosinase bound to BMN11. (b) The black lines show a magnified image of the BMN11-tyrosinase binding site. The binding residues of (c) kojic acid and (d) BMN11 with tyrosinase were analyzed using LigandScout 3.1 software.

Because tyrosinase is an essential factor for melanin synthesis [19], we further investigated whether BMN11 can directly bind to tyrosinase to inhibit its activity by docking simulation. We used Dock6 to simulate the interaction between BMN11 and tyrosinase. The protein structure images showed that BMN11 binds to tyrosinase (Figures 5a-5b). The binding affinity of kojic acid to tyrosinase was -27.7 kcal/mol, whereas that of BMN11 was -30.45 kcal/mol (Table 3), indicating that BMN11 may bind to tyrosinase with stronger affinity than kojic acid does.

**Table 2:** V_max_ and K_m_ values for BMN11

Concentration (μM)	V_max_	K_m_
NO inhibitor	2.93 X 10^-2^	0.73
BMN11 10	2.95 X 10^-2^	1.20
BMN11 50	2.96 X 10^-2^	1.61

**Table 3:** Tyrosinase docking score for BMNs

Compounds	Tyrosinase Docking Score
BMN1	-27.48
BMN2	-27.86
BMN3	-28.11
BMN4	-27.21
BMN5	-30.13
BMN6	-26.86
BMN7	-30.37
BMN8	-29.44
BMN9	-30.84
BMN10	-26.82
BMN11	-30.45
BMN12	-28.34
Kojic Acid	-27.7
Arbutin	-33.05
Resveratrol	-29.52
Kaempferol	-30.39
Cinnamic acid	-24.78

We used the LigandScout 3.1 software to examine the binding residues of tyrosinase that interact with BMN11 or kojic acid. Kojic acid can bind to tyrosinase mainly via an aromatic interaction with HIS263 and a hydrogen bond with the MET280 residue of tyrosinase (Figure 5c). BMN11 forms two hydrogen bonds with the GLY281 and ASN260 residues of tyrosinase and three hydrophobic interactions with the VAL283, PHE264, and ALA286 residues of tyrosinase (Figure 5d), and this likely contributes to the higher inhibitory effect of BMN11 on tyrosinase.

**Figure 6:**
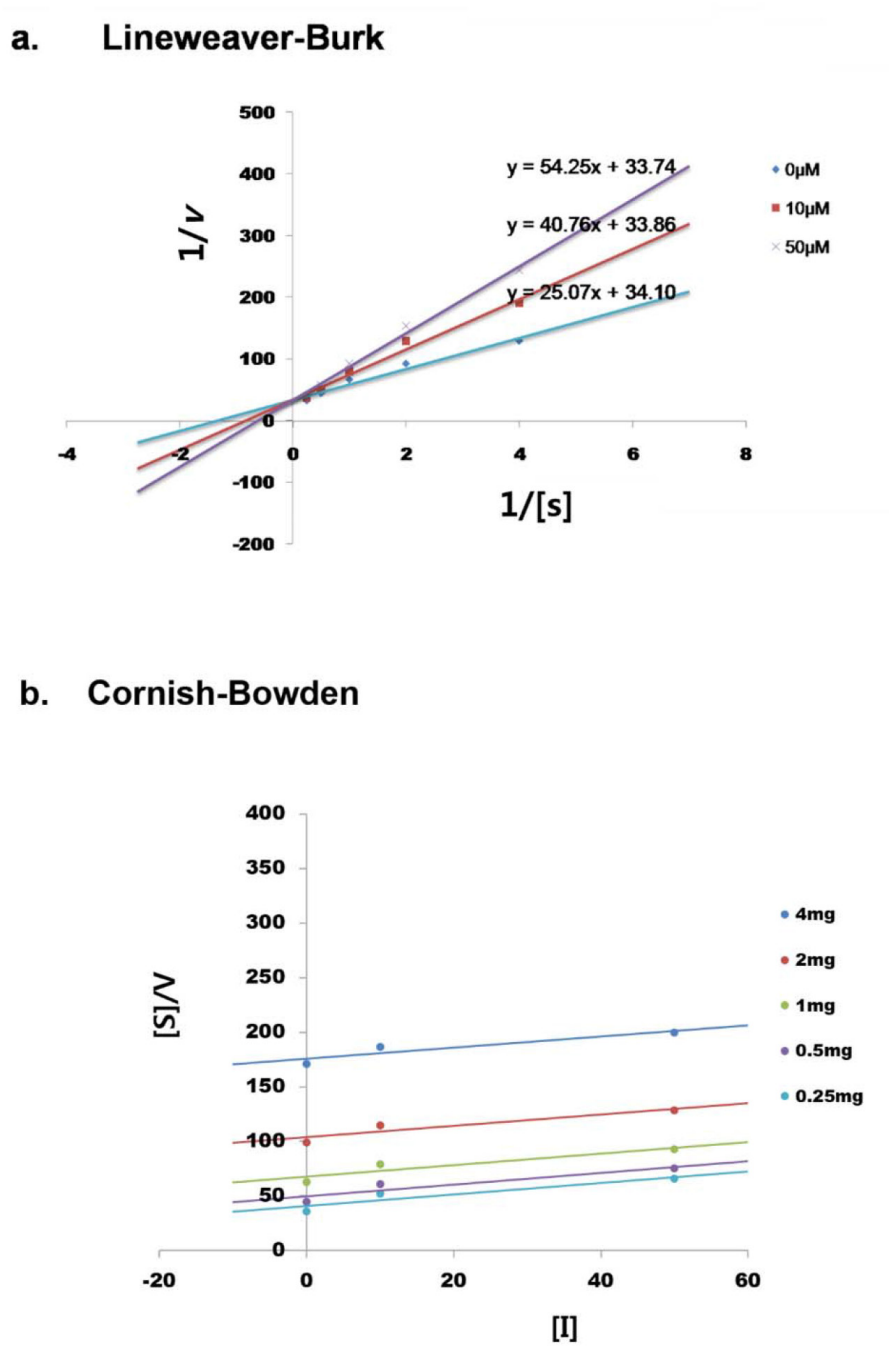
BMN11 as a competitive inhibitor of tyrosinase **(a)** Lineweaver–Burk plot analysis of mushroom tyrosinase was performed using BMN11. Data were obtained as mean values of 1/V (the inverse of the increase in absorbance at a wavelength of 492 nm per min) of three independent tests with different concentrations of *L-DOPA* as a substrate. X axis= 1/[*L-DOPA*], mM^-1^, Y axis= 1/*v* (∆492/min)^-1^. **(b)** Cornish-Bowden plot analysis of the tyrosinase inhibitory effect of BMN11. Data were obtained as mean values of [S]/V. [I] represents BMN11. X axis= [BMN11], μM^-1^, Y axis= [S]/V = 1/Vmax ([S] + Km) + Km/ (Vmax·Ki).

Based on the docking simulation and binding residue analysis, we assumed that BMN11 might competitively bind to tyrosinase with tyrosine. Thus, we performed a Lineweaver-Burk analysis (Figure 6a) to examine the mode of inhibition by BMN11. As the concentration of BMN11 was increased, K_m_ values also gradually increased while V_max_ values were unchanged (Table 2). Cornish-Bowden analysis showed that the slopes at different concentrations of BMN11 are parallel (Figure 6b), indicating that BMN11 is a competitive inhibitor of tyrosinase.

**Figure 7:**
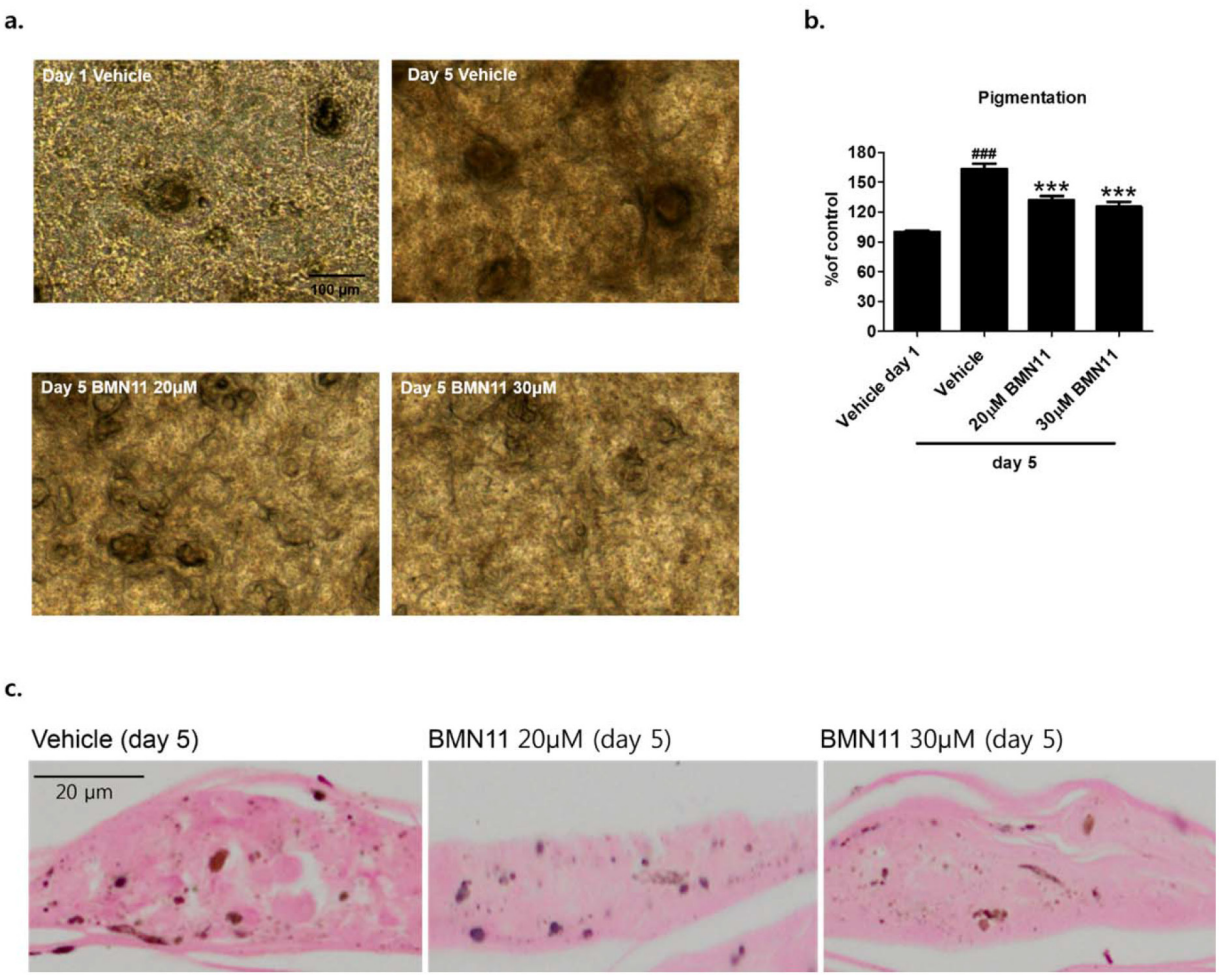
Inhibition of melanin accumulation by BMN11 in a human skin model The human skin model was treated with DMSO (vehicle) or BMN11 and cultured in the maintenance media provided by the company for 5 d. **(a)** Representative microscopic images of the human skin model with or without BMN11 treatment. **(b)** The darkness of the skin was analyzed by image J software. **(c)** Representative images of Fontana-Masson staining showing the epidermis of the human skin model. Each value was expressed as mean ± SEM. Data were analyzed using one-way ANOVA followed by Bonferroni test. ^###^p < 0.001 vs. the day 1 group without any treatment, ***p < 0.001 vs. the day 5 group (control treated with DMSO).

To further confirm that BMN11 suppresses melanogenesis in human, we cultured a viable, reconstituted, three dimensional human epidermis consisting of human melanocytes and keratinocytes (Neoderm^®^-ME, Tegoscience Co) that has been shown to undergo spontaneous melanogenesis with time [20]. The human skin model was treated with BMN11 and cultured in the maintenance media (Tegoscience Co) for 5 d. Compared with the day 1, the color of the human skin model was darkened at day 5 and BMN11 treatment brightened it (Figure 7a). Consistently, Fontana-Masson staining revealed that melanin level in the epidermis was decreased by BMN11 (Figure 7b), suggesting that BMN11 is also effective for the suppression of melanogenesis in the human skin model. However, BMN11 treatment at 30μM appears not to additively inhibit skin pigmentation compared to the BMN11 treatment at 20μM (Figures 7b-7c). Thus, we assume that BMN11 at 20μM may be enough for the maximal anti-melanogenic effect of BMN11 in the human skin model.

## DISCUSSION

In an effort to find better tyrosinase inhibitors, we synthesized 12 new 2-(substituted benzylidene) malononitrile derivatives and investigated their anti-melanogenic activities. Of these, BMN11 exhibited the strongest tyrosinase inhibitory activity. Consistent with this, BMN11 markedly inhibited αMSH-induced melanogenesis without cytotoxicity in B16F10 cells and suppressed melanin accumulation in the human skin model. Although more studies are necessary to reveal the mechanism underlying BMN11-mediated inhibition of tyrosinase, it appears that instead of binding to tyrosine, BMN11 may bind directly to tyrosinase by forming two hydrogen bonds with the GLY281 and ASN260 residues of tyrosinase, and via three hydrophobic interactions with the VAL283, PHE264, and ALA286 residues of tyrosinase, which partially explains the stronger inhibitory activity of BMN11 against tyrosinase than that of kojic acid.

Based on the results of our preliminary experiments (unpublished data) and those of another study [21], many 3, 4-dihydroxyphenyl compounds were reported to exhibit weak tyrosinase inhibitory activity. However, the inhibitory activities of 3, 4-dihydroxyphenyl compounds can be increased based on the chemical structure attached to the 3, 4-dihydroxyphenyl moiety. As an example, our previous study showed that MHY-794 ((±)-4-(thiazolidin-2-yl)benzene-1, 2-diol), a 3, 4-dihydroxyphenyl compound, exhibited excellent tyrosinase inhibitory activity in mushroom tyrosinase and cell-based tyrosinase assays [22]. More studies are necessary to reveal how certain chemical structures attached to the 3, 4-dihydroxyphenyl moiety are able to regulate tyrosinase activity.

When the docking scores are compared, the predicted binding affinities of BMN5 (-30.13 kcal/mol), BMN7 (-30.37 kcal/mol), and BMN9 (-30.84 kcal/mol) are comparable to that of BMN11 (-30.45). However, BMN5, BMN7, and BMN9 do not inhibit tyrosinase activity. Although it is hard to explain the discrepancy based on our data, it is likely that other BMNs may not easily locate the binding pocket of tyrosinase in the experiment using mushroom tyrosinase unlike the results from the protein-ligand docking simulation, in which the site of the binding pocket are artificially designated. Also, it is unclear how BMN11 of many similar compounds exhibites the strongest inhibitory activity against tyrosinase. According to our accumulated structure-activity relationship (SAR) data (unpublished), the 2, 4-dihydroxyphenyl moiety has played a very important role in showing potent tyrosinase inhibitory activity in many cases. Thus, the synthesis of BMN analog with a 2, 4-dihydroxyphenyl moiety was attempted using the same reaction conditions (a catalytic amount of NaOH, EtOH/water(1:4), 55 °C) used for the synthesis of BMN1 – BMN12, but the desired product was not obtained. Our accumulated SAR data also indicate a tendency to decrease tyrosinase inhibitory activity in the following order: 2, 4-dihydroxyphenyl > 3-hydroxy-4-methoxyphenyl > 3, 4-dihydroxyphenyl = 4-hydroxyphenyl = 4-hydroxy-3-methoxyphenyl >> other phenyls. However, the tendency is not strict and relies on the structure of the remaining residues of the compound. As expected, BMN11 having a 3, 4-dihydroxyphenyl substituent exerted strong tyrosinase inhibition, but compounds with 4-hydroxyphenyl (BMN1), 4-hydroxy-3-methoxyphenyl (BMN2) and 3-hydroxy-4-methoxyphenyl (BMN3) substituents showed only moderate to weak tyrosinase inhibition. Similarly, in our SAR data, only one or two compounds with the above-mentioned substituents in the phenyl substituted compounds exhibit strong inhibitory activity against tyrosinase, while the remaining compounds with the indicated substituents show only moderate or weak inhibition. Our SAR data also reveal that the presence of at least one hydroxyl group on the phenyl ring is essential for strong tyrosinase inhibition.

Although our study shows that BMN11 is a tyrosinase inhibitor to prevent skin pigmentation, it is not tested whether BMN11 is actually absorbed through keratinocytes to exhibit the anti-melanogenic effect. Previously, we reported that two synthetic diphenols, 3-DBP [23] and MHY-794 [22], exhibited anti-melanogenic effects *in vivo* through tyrosinase inhibition. The inhibitory effect of melanin formation *in vivo* implied that these diphenol compounds are absorbed through keratinocytes. The log P values for 3-DBP and MHY-794 calculated using the ChemDraw Ultra 12.0 program were 0.12 and 1.59, respectively. Therefore, it is expected that the diphenol compound having log P = 1 or more is absorbed sufficiently through keratinocytes. The calculated log P value of BMN11 by the program was 1.53, so that BMN11 is expected to be absorbed through keratinocytes. In addition, the anti-melanogenic effect of BMN11 on the human skin model indirectly shows that BMN11 can be absorbed through keratinocytes.

Unfavorable side effects are major concerns in many synthetic inhibitors against tyrosinase. Although our study did not provide conclusive evidence about safety of BMN11 for its application to human skin, BMN11 did not show cytotoxicity in cell lines of HaCat (human keratinocyte), B16F10 (mouse melanoma), Hs27 (human fibroblast) in our experimental conditions. Furthermore, when BMN11 was treated to the human skin model with the concentrations that did not show cytotoxicity in the cell lines, there were no visible signs of toxicity such as cell detachment or cell debris formation based on microscopic observation. However, there are few studies available that examine toxicity of 2-(Substituted benzylidene)malononitrile derivatives for its application to human skin, further *in vivo* studies are necessary to test its safety in physiology.

In conclusion, our study proved in cell and human skin models that BMN11 is the strongest tyrosinase inhibitor of the 12 2-(substituted benzylidene) malononitrile derivatives tested and can be used as an anti-melanogenic compound in cosmetics that aim to have whitening effects on skin.

## MATERIALS AND METHODS

### Synthesis of compounds

#### General synthetic method for BMN1 – BMN12

Synthesis of BMN1 – BMN11: NaOH (5 mg, 0.13 mmol) was added to a stirred solution of malononitrile (145 mg, 2.19 mmol) and an appropriate substituted benzaldehyde (0.91 equivalent) in ethanol (5 mL) and water (20 mL). The reaction mixture was heated to 55 °C for 15 min – 1 h (see data of each product and Figure 1). After cooling, the reaction mixture was neutralized with aqueous ammonium chloride solution (10 mL). The precipitates generated during the addition of aqueous ammonium chloride solution were filtered and the filter cake was washed with water (30 mL) and ethyl alcohol (5 mL) to remove remaining starting materials and by-products, producing BMN1 – BMN11 as solids with 35 – 92% yields (see data of each product). BMNs were relatively insoluble in EtOH and water co-solvent, but the remaining starting materials and by-products were relatively easily dissolved in EtOH and water. Therefore, no additional purification steps were required other than filtration and washing with EtOH and water.

Synthesis of BMN12: A solution of malononitrile (145 mg, 2.19 mmol) in water (10 mL), and subsequently NaOH (5 mg, 0.13 mmol), was added to a stirred solution of 3, 5-dibromo-4-hydroxybenzaldehyde (275 mg, 1.99 mmol) in 1, 4-dioxane (2 mL) and ethyl alcohol (10 mL). The reaction mixture was heated to 55 °C for 6 h. After cooling, the reaction mixture was neutralized with aqueous ammonium chloride solution (10 mL). The precipitates generated during the addition of aqueous ammonium chloride solution were filtered and the filter cake was washed with water (30 mL) and ethyl alcohol (5 mL) to remove remaining starting materials and by-products, producing BMN12 as a solid with 62% yield.

NMR analysis:^1^H and ^13^C nuclear magnetic resonance (NMR) spectra were recorded on Varian Unity Inova 400 and Varian Unity AS 500 instruments (Agilent Technologies, Santa Clara, CA, USA). Chemical shifts were reported with reference to the respective residual solvent or deuterated solvent peaks (*δ*_H_ 2.49 and *δ*_C_ 39.7 for DMSO-*d*_6_).

#### 2-(4-Hydroxybenzylidene)malononitrile (BMN1)

Reaction time, 1 h; yield, 74%; ^1^H NMR (400 MHz, DMSO-*d*_6_) *δ* 11.04 (brs, 1 H, OH), 8.28 (s, 1 H, vinylic H), 7.87 (d, 2 H, *J =* 8.0 Hz, 2′-H, 6′-H), 6.95 (d, 2 H, *J =* 8.0 Hz, 3′-H, 5′-H); ^13^C NMR (100 MHz, DMSO-*d*_6_) *δ* 164.6, 161.2, 134.6, 123.5, 117.3, 115.8, 114.9, 75.7.

#### 2-(4-Hydroxy-3-methoxybenzylidene)malononitrile (BMN2)

Reaction time, 1 h; yield, 80%; ^1^H NMR (400 MHz, DMSO-*d*_6_) *δ* 10.77 (brs, 1 H, OH), 8.25 (s, 1 H, vinylic H), 7.61 (d, 1 H, *J =* 2.0 Hz, 2′-H), 7.48 (dd, 1 H, *J =* 8.4, 2.0 Hz, 6′-H), 6.96 (d, 1 H, *J* = 8.4 Hz, 5′-H), 3.78 (s, 3 H, OCH_3_); ^13^C NMR (100 MHz, DMSO-*d*_6_) *δ* 161.3, 154.5, 148.6, 128.4, 123.7, 116.8, 115.8, 115.0, 113.7, 75.6, 56.2.

#### 2-(3-Hydroxy-4-methoxybenzylidene)malononitrile (BMN3)

Reaction time, 1 h; yield, 83%; ^1^H NMR (500 MHz, DMSO-*d*_6_) *δ* 9.81 (brs, 1 H, OH), 8.26 (s, 1 H, vinylic H), 7.51 (s, 1 H, 2′-H), 7.42 (d, 1 H, *J =* 8.5 Hz, 6′-H), 7.14 (d, 1 H, *J =* 8.5 Hz, 5′-H), 3.87 (s, 3 H, OCH_3_); ^13^C NMR (100 MHz, DMSO-*d*_6_) *δ* 161.4, 154.5, 147.6, 127.1, 125.0, 115.9, 115.7, 114.6, 112.9, 76.9, 56.7.

#### 2-(3-Ethoxy-4-hydroxybenzylidene)malononitrile (BMN4)

Reaction time, 1 h; yield, 92%; ^1^H NMR (500 MHz, DMSO-*d*_6_) *δ* 10.70 (brs, 1 H, OH), 8.24 (s, 1 H, vinylic H), 7.61 (s, 1 H, 2′-H), 7.46 (d, 1 H, *J* = 8.5 Hz, 6′-H), 6.97 (d, 1 H, *J* = 8.5 Hz, 5′-H), 4.03 (q, 2 H, *J* = 7.0 Hz, C*H*_*2*_CH_3_), 1.35 (t, 3 H, *J* = 7.0 Hz, CH_2_C*H*_*3*_); ^13^C NMR (100 MHz, DMSO-*d*_6_) *δ* 161.3, 154.7, 147.7, 128.4, 123.7, 116.8, 115.8, 115.0, 114.6, 75.5, 64.5, 15.1.

#### 2-(3, 4-Dimethoxybenzylidene)malononitrile (BMN5)

Reaction time, 1 h; yield, 35%; ^1^H NMR (500 MHz, DMSO-*d*_6_) *δ* 8.33 (s, 1 H, vinylic H), 7.62 (d, 1 H, *J* = 1.5 Hz, 2′-H), 7.59 (dd, 1 H, *J* = 8.5, 1.5 Hz, 6′-H), 7.20 (d, 1 H, *J* = 8.5 Hz, 5′-H), 3.87 (s, 3 H, OCH_3_), 3.78 (s, 3 H, OCH_3_); ^13^C NMR (100 MHz, DMSO-*d*_6_) *δ* 161.3, 155.1, 149.4, 128.0, 124.8, 115.5, 114.8, 112.7, 112.5, 77.3, 56.7, 56.1.

#### 2-(4-Methoxybenzylidene)malononitrile (BMN6)

Reaction time, 30 min; yield, 59%; ^1^H NMR (500 MHz, DMSO-*d*_6_) *δ* 8.37 (s, 1 H, vinylic H), 7.96 (d, 2 H, *J* = 9.0 Hz, 2′-H, 6′-H), 7.17 (d, 2 H, *J* = 9.0 Hz, 3′-H, 5′-H), 3.87 (s, 3 H, OCH_3_); ^13^C NMR (100 MHz, DMSO-*d*_6_) *δ* 165.0, 161.2, 134.1, 124.8, 115.9, 115.5, 114.6, 77.5, 56.6.

#### 2-(3, 4, 5-Trimethoxybenzylidene)malononitrile (BMN7)

Reaction time, 40 min; yield, 62%; ^1^H NMR (400 MHz, DMSO-*d*_6_) *δ* 7.65 (s, 1 H, vinylic H), 7.18 (s, 2 H, 2′-H, 6′-H), 3.97 (s, 3 H, OCH_3_), 3.90 (s, 6 H, 2XOCH_3_); ^13^C NMR (100 MHz, DMSO-*d*_6_) *δ* 159.7, 153.6, 144.2, 126.2, 114.3, 113.5, 108.5, 80.8, 61.5, 56.6.

#### 2-(4-Hydroxy-3, 5-dimethoxybenzylidene)malononitrile (BMN8)

Reaction time, 30 min; yield, 86%; ^1^H NMR (400 MHz, DMSO-*d*_6_) *δ* 8.20 (s, 1 H, vinylic H), 7.35 (s, 2 H, 2′-H, 6′-H), 3.78 (s, 6 H, 2XOCH_3_); ^13^C NMR (100 MHz, DMSO-*d*_6_) *δ* 161.2, 148.6, 144.2, 122.2, 115.9, 115.2, 109.7, 75.5, 56.7.

#### 2-(2, 4-Dimethoxybenzylidene)malononitrile (BMN9)

Reaction time, 30 min; yield, 61%; ^1^H NMR (400 MHz, DMSO-*d*_6_) *δ* 8.23 (s, 1 H, vinylic H), 8.01 (d, 1 H, *J* = 9.2 Hz, 6′-H), 6.75 (dd, 1 H, *J* = 8.8, 2.4 Hz, 5′-H), 6.70 (d, 1 H, *J* = 2.4 Hz, 3′-H), 3.88 (s, 3 H, OCH_3_), 3.88 (s, 3 H, OCH_3_); ^13^C NMR (100 MHz, DMSO-*d*_6_) *δ* 167.5, 162.0, 154.5, 131.2, 116.0, 114.9, 113.8, 108.4, 99.0, 76.5, 57.0, 56.8.

#### 2-(3-Bromo-4-hydroxybenzylidene)malononitrile (BMN10)

Reaction time, 15 min; yield, 65%; ^1^H NMR (400 MHz, DMSO-*d*_6_) *δ* 8.23 (s, 1 H, vinylic H), 8.01 (d, 1 H, *J* = 2.0 Hz, 2′-H), 7.87 (dd, 1 H, *J* = 8.8, 2.0 Hz, 6′-H), 7.12 (d, 1 H, *J* = 8.4 Hz, 5′-H); ^13^C NMR (100 MHz, DMSO-*d*_6_) *δ* 160.8, 159.9, 137.1, 132.7, 124.7, 117.7, 115.4, 114.4, 111.0, 77.8.

#### 2-(3, 4-Dihydroxybenzylidene)malononitrile (BMN11)

Reaction time, 20 min; yield, 72%; ^1^H NMR (500 MHz, DMSO-*d*_6_) *δ* 10.02 (brs, 2 H, 2XOH), 8.17 (s, 1 H, vinylic H), 7.52 (d, 1 H, *J* = 1.0 Hz, 2′-H), 7.32 (d, 1 H, *J* = 8.5, 1.0 Hz, 6′-H), 6.90 (d, 1 H, *J* = 8.5 Hz, 5′-H); ^13^C NMR (100 MHz, DMSO-*d*_6_) *δ* 161.3, 154.0, 146.7, 127.7, 123.9, 116.8, 116.6, 116.0, 114.9, 75.0.

#### 2-(3, 5-Dibromo-4-hydroxybenzylidene)malononitrile (BMN12)

Reaction time, 6 h; yield, 62%; ^1^H NMR (500 MHz, DMSO-*d*_6_) *δ* 8.24 (s, 1 H, vinylic H), 8.14 (s, 2 H, 2′-H, 6′-H); ^13^C NMR (100 MHz, DMSO-*d*_6_) *δ* 158.3, 157.6, 135.6, 125.3, 115.2, 114.2, 112.9, 79.2.

### Docking simulation of tyrosinase and BMNs

Dedicator of cytokinesis 6 (Dock6) was used for the *in silico* protein–ligand docking simulation. The results from Dock6 were confirmed using the AutoDock4.2 program. The 3D structure of tyrosinase was used in the crystal structure of *Agaricus bisporus* (PDB ID: 2Y9X). The predefined binding site of tyrosine was used as a docking pocket. The following steps were performed to prepare the structures of compounds for docking simulation, (1) 2D structures were converted into 3D structures, (2) charges were calculated, and (3) hydrogen atoms were added using the ChemOffice program (http://www.cambridgesoft.com). Afterwards, docking simulations between tyrosinase and BMN11 or kojic acid were performed. The predicted hydrogen bonding residues between different compounds and tyrosinase, and generation of pharmacophores were investigated with the LigandScout 3.0 software.

### Tyrosinase activity assay using mushroom tyrosinase

BMN 1∼12 (50 μM) and kojic acid (50 μM) were loaded onto a 96-well microplate (Nunc, Denmark) in tyrosinase buffer (200 μL) containing 1 mM l-tyrosine solution, 50 mM phosphate buffer (pH 6.5), and mushroom tyrosinase (1000 U) [24]. The plate was incubated at 37°C for 15 min. Dopaquinone was measured by spectrophotometry at 450 nm. Based on this, the IC_50_ was calculated using log-linear curves and their equations.

### Kinetic analysis of tyrosinase inhibition by BMN11

l-Tyrosine was prepared at concentrations of 16, 8, 4, 2, 1, 0.5, 0.25, 0.125, 0.0625, and 0.03125 mM, and BMN11 was prepared at 2, 5, and 10 μM. Reaction mixture solution was prepared in a 96-well plate, in which 20 μL of tyrosinase substrate (l-tyrosine), 10 μL of an aqueous mushroom tyrosinase solution (200 U), and 50 mM potassium phosphate buffer (pH 6.5) were added. The dopachrome production rate of the reaction mixture was measured at a wavelength of 450 nm using a microplate reader. The tyrosinase inhibition rate of BMN11 was then determined using Lineweaver-Burk plot analysis. The Michaelis constant (K_m_) and maximal velocity (V_max_) were also calculated by Lineweaver-Burk plots with different concentrations of l-tyrosine substrate [25].

### Cell culture and viability assay

B16F10 melanoma cells were purchased from the Korea Cell Line Bank. The cells were cultured in Dulbecco’s modified Eagle’s medium (DMEM) with 5% fetal bovine serum (FBS), and 1% penicillin, streptomycin, l-glutamine, and sodium pyruvate. The cells were maintained at 37°C in a humidified 95% air/5% CO_2_ atmosphere. For the cell viability assay, cells were seeded in 96-well plates. B16F10 melanoma cells were treated with BMN11 at various concentrations for 48 h. Ez-Cytox (10 μL) was added to each well and incubated for 2 h. The formazan crystals formed was measured by spectrophotometry at 450 nm. Cell viability was calculated using cells without BMN11 treatment as the control group.

### Melanin content in B16F10 cells

Melanin concentration was determined using the method previously described with slight modifications [26]. B16F10 cells were seeded in 6-well plates and allowed to grow to 70-80% confluence. The cells were pre-treated with BMN11 or kojic acid for 2 h. After this, αMSH was added to the BMN11- or kojic acid-containing medium and incubated for further 48 h. After washing with PBS, the cells were detached using trypsin and dissolved in 90 μL 1N NaOH solution containing DMSO (5%). After incubation at 60°C for 1 h, melanin content was determined by measuring absorbance at 405 nm.

### Melanin accumulation in a human skin model

A viable, reconstituted, three dimensional human epidermis (Neoderm-ME, Tego Science) was used to examine the anti-melanogenic effect of BMN11 in a human skin model. The human skin model was pretreated with DMSO (vehicle) or BMN11 for 1 h and cultured in the maintenance media provided by the company for 5 d with DMSO or BMN11 treatment. Microscopic analysis was performed at day 1 to day 5 to observe skin pigmentation. The microscopic images were analyzed by image J software to semi-quantify the darkening of the skin. For Fontana-Masson staining, skin samples were fixed in 4% paraformaldehyde overnight at room temperature and the samples were analyzed by a commercially available company (Garam Meditech, South Korea).

### Statistical analysis

All data are presented as mean ± SEM. Different groups were compared using one-way analysis of variance followed by the Bonferroni post-test. *P* < 0.05 was considered statistically significant.
